# Estimated 24-hour urinary sodium excretion as a risk factor for oxidative stress in Zambian adults: A cross-sectional study

**DOI:** 10.1371/journal.pone.0242144

**Published:** 2020-11-12

**Authors:** Violet Kayamba, Paul Kelly

**Affiliations:** 1 Tropical Gastroenterology & Nutrition Group, Department of Internal Medicine, University of Zambia School of Medicine, Lusaka, Zambia; 2 Blizard Institute, Barts & The London School of Medicine and Dentistry, Queen Mary University of London, London, United Kingdom; International University of Health and Welfare, School of Medicine, JAPAN

## Abstract

**Introduction:**

Persistent oxidative stress predisposes to various non-communicable diseases (NCDs), whose occurrence is increasing in sub-Saharan Africa. The aim of this study was to evaluate the link between markers of oxidative stress and some risk factors for NCDs in a Zambian cohort.

**Methods:**

We assessed oxidative stress by measuring 8-isoprostane (lipid oxidative stress) and 8-hydroxydeoxyguanosine (DNA oxidative stress). In addition, we measured mycotoxins (aflatoxin M1 and ochratoxin A), salt intake estimated from 24-hour sodium excretion calculated using the Tanaka and Kawaski formulae, and 1-hydroxypyrene (a metabolite of polycyclic aromatic hydrocarbons). Data on lifestyle risk factors were collected using questionnaires.

**Results:**

Included were 244 participants; 128 (52%) were female and the median age was 48 years (IQR 39–58). The median level of 8-isoprostane was 0.13 ng/mg creatinine (IQR 0.08–0.23) while that of 8-hydroxydeoxyguanosine (8-OHdG) was 4 ng/mg creatinine (IQR 2–10). The median 24-hour sodium excretion was 21 g (IQR 16–25 g), with none being less than the 5 g recommended by WHO. Unadjusted urinary levels of 8-isoprostane were moderately correlated with 1-hydroxypyrene (Spearman r = 0.30, *p*<0.001) and estimated 24-hour urine sodium (Spearman r = 0.38, *p*<0.001). Urinary levels of 8-OHdG were not correlated with 1-hydroxypyrene, estimated 24-hour urine sodium, aflatoxin M1 or ochratoxin A (all *p*-values >0.05). Using logistic regression, adjusted and unadjusted 8-isoprostanes levels were associated with 1-hydroxypyrene (*p* = 0.02 and *p* = 0.001 respectively) and estimated 24-hour urine sodium method (*p* = 0.003 and *p*<0.001 respectively). However, only unadjusted 8-OHdG was associated with 1-hydroxypyrene (*p* = 0.03) and age (*p* = 0.007).

**Conclusions:**

Estimated 24-hour urinary sodium is high among Zambians and it is associated with lipid but not DNA oxidative stress. High exposure to polycyclic aromatic hydrocarbons is also associated with oxidative stress.

## Background

Sub-Saharan Africa has a large and growing burden of non-communicable disease (NCD) [1]. Long-term oxidative stress is known to contribute to the occurrence of NCDs such as cancer, diabetes mellitus, cardiovascular, renal and neurodegenerative diseases [2]. Oxidative stress results from a disturbance in the balance between reactive oxygen species (ROS) or free radicals and antioxidant defence mechanisms such as glutathione-dependent peroxidases and superoxide dismutase [3]. ROS when maintained at physiological levels are involved in a number of cellular signalling pathways and immune defences [4]. Disturbance of this balance leads to tissue or cellular damage, predisposing the host to various disease states.

The presence of oxidative stress can be established directly by measuring ROS, or indirectly by measuring molecules generated by oxidative damage to DNA, lipids or proteins [5]. Among the products of oxidative degradation of lipids are isoprostanes, prostaglandin-like compounds produced by the reaction of free radicals with arachidonic acid. They are stable products detectable in body fluids, and can thus be measured as biomarkers of oxidative stress [5, 6]. Oxidative damage to DNA causes alterations in the bases, and guanosine is especially susceptible to oxidation. 8-hydroxydeoxyguanosine is therefore, utilized as a biomarker of oxidative DNA damage [5, 7].

Oxidative stress can be triggered by dietary and environmental factors [8, 9]. High salt (sodium chloride) intake is a dietary factor that has been shown to enhance the generation of ROS and suppress the activity of the superoxide dismutase (an enzyme that scavenges the superoxide radicals) in animal models [10, 11]. There is a paucity of direct evidence linking high salt intake and oxidative stress in humans [12] with most studies looking at the effects indirectly. It is however, known that low salt intake is associated with a reduction of all-cause mortality, cardiovascular disease, kidney disease, gastric cancer and osteoporosis [11]. The World Health Organisation (WHO) recommends an intake of salt not more than 5g per day, but most people consume more than twice this limit [13]. It is estimated that about 2.5 million deaths per year could be prevented if global salt consumption were reduced to levels recommended by the WHO.

Available data show that the average intake of salt in sub-Saharan Africa is much higher than the WHO recommended maximum [13, 14]. Determining the exact amounts of salt people consume is practically challenging. Therefore 24-hour urine sodium, despite its limitations, is used to estimate salt consumption on the assumption that input equals output in the steady state. In epidemiological studies, 24-hour urine sodium has been calculated using spot urine samples [15–18]. A good correlation between measured and estimated 24-hour urine sodium has been reported [19], although mis-classifications are frequent if measured on an individual basis [20].

Inhalation or ingestion of environmental contaminants can cause oxidative stress. Mycotoxins are one of the most common contaminants of staple foods in Africa [21, 22], and their exposure is almost ubiquitous in some rural communities [23]. Mycotoxins such as aflatoxins, are produced by fungi under certain conditions and their consumption may lead to various acute and chronic conditions including cancer [24]. Polycyclic aromatic hydrocarbons (PAH) are organic compounds found in environmental contaminants such as biomass smoke [25]. PAHs are lipophilic and hence easily absorbed with high bioavailability when ingested or inhaled. Such exposures are difficult to measure objectively, but indirect methods determining the presence of known metabolites can used. One such metabolite is 1-hydroxyprene, which is a product of PAH metabolism [26].

In this study, we evaluated the association between biomarkers of oxidative stress (8-isoprostane and 8- hydroxydeoxyguanosine) and some known risk factors for non-communicable disease (salt, mycotoxins and PAH).

## Methods

### Recruitment of study participants

This was a cross-sectional study conducted at the University Teaching Hospital in Lusaka, Zambia. We studied participants who had been used as healthy controls in a previous gastric cancer study [27]. Only those who gave written consent were included in this study. Interviewer administered questionnaires were used to collect basic demographic data. Participant’s weight (kg) and height (m) were measured. Blood and spot urine samples were collected from each study participant and stored at -80°C prior to further analysis.

Testing for Human Immunodeficiency Virus (HIV) was done using Uni-Gold™ rapid diagnostic kits (Trinity Biotech, Wicklow, Ireland). The University of Zambia Biomedical Research Ethics committee, reference number 000-03-16, approved the study.

### Evaluation of markers of oxidative stress: Urinary 8-isoprostane and 8-hydroxydeoxyguanosine

Enzyme linked immunosorbent assay (ELISA; Detroit R&D, Detroit, USA) was used to measure urine levels of 8-isoprostane, following the manufacturer’s instructions. The readings were corrected for creatinine, and the final results reported as ng of 8-isoprostanes per mg of creatinine. Urine levels of 8-hydroxydeoxyguanosine (8-OHdG) were also measured by ELISA (MyBioSource, San Diego, USA) following the manufacturer’s instructions. Final results were corrected for creatinine excretion and reported as ng 8-OHdG per mg of creatinine.

### Measurement of urinary 1-hydroxypyrene

High performance liquid chromatography (HPLC) with fluorescence detection for 1-hydroxypyrene (1-OHP) was conducted as reported previously [27]. Briefly, the Phenolic Compound stock standard (Clincal^R^) ref. no. 9925 (Recipe), was used as the calibrator. To all samples, calibrators and controls β-glucoronidase enzyme mix was added. The % recovery of the two certified reference controls Clincheck Level 1 and 2 were 97% and 95%, respectively. The limit of quantitation was 0.052 μg/l. A “Waters” system HPLC was used, with a Binary Pump (1525), Autosampler (717), multi and a fluorescence detector (2475). The excitation wavelength was set at 242 nm and the emission wavelength at 388 nm. The flow rate was 1ml/min for 5 minutes. Results were reported as μg per g creatinine.

### Measurement of urinary aflatoxin M1 and serum ochratoxin A

Urine levels of aflatoxin M1 were measured by ELISA (Helica Biosystems, Santa Ana, USA). Obtained values were corrected for creatinine to derive excretion values in ng of aflatoxin M1 per mg of creatinine. Similarly, ochratoxin A levels were measured in serum by ELISA (Helica Biosystems, Santa Ana, USA). Results were reported as ng per ml of serum.

### Estimated 24-hour urine sodium excretion

To calculate the estimated 24-hour urine sodium excretion, the Tanaka [15] and Kawasaki [16, 17] formulae were used. Standard chemistry procedures were used to determine the levels of sodium and creatinine in the spot urine samples collected.

#### Tanaka method

This method is based on the patient’s weight, age and height for both sexes. To estimate the 24-hour urine sodium (Na24h) from a spot sample, the 24-hour creatinine excretion (CrPr24h) was calculated as follows:

*CrPr24h (mg) = [(14*.*89* *x weight*, *kg) + (16*.*14 x height*, *cm) (2*.*04 x age*, *years)]– 2*,*244*,*45*.

The Na24h (mEq) excretion is then estimated as *NaUr (mEq) = 21*.*98 x [Na casual urine*, *mEq/L/(Cr casual urine*, *mg/dL x 10)] x CrPr24 h (mg)*.

#### Kawasaki method

This method is also based on the patient’s weight, age and height but the formulae are different for each of the sexes.

For males the estimation for CrPr24h was:

*CrPr24h (mg) = [(15*.*12 x weight*, *kg) + (7*.*39 x height*, *cm) (12*.*63 x age*, *years)]– 79*.*9*.

For females the estimation for CrPr24h was:

*CrPr24h (mg) = [(8*.*58 x weight*, *kg) + (5*.*09 x height*, *cm) (4*.*72 x age*, *years)– 74*.*95*.

The Na24h (mEq) excretion is then estimated as *NaUr (mEq) = 16*.*3 X (√ [(Na casual urine (mEq/L)/(Cr casual urine mg/dL X 10)] x CrPr24h (mg)) x (CrPr24h)]*.

### Self reported salt consumption by study participants

To inquire about salt intake and preference, participants were asked if they added salt to already prepared food with ordinal responses including “all the time”, “very often”, “not often” or “never”. On salt preference, participants were asked to choose the most applicable response for them, “not salty”, “slightly salty”, “salty” or “very salty”.

### Statistical analysis

Continuous variables were checked for normality using the Shapiro-Wilk test and summarised using medians and interquartile ranges as they were all non-parametric. To determine if there were any statistically significant differences between two or more groups of independent variables on continuous or ordinal variables, we used the Kruskal-Wallis test. To test for correlations, Spearman's rank correlation coefficient was used. In addition, a stepwise logistic regression analysis was employed to access the relative contributions of different exposure variables. In all cases, a two-sided *p* value less than 0.05 was considered statistically significant. Statistical analysis was done in STATA version 15 (College Station, TX, USA).

## Results

### Basic characteristics of study participants in relation to markers of oxidative stress

We enrolled 244 study participants; 128 (52%) were female. The median age was 48 years (IQR 39–58). Eighty two percent of the participants were from urban areas and 16 (7%) had no formal education (Table 1). The median level of unadjusted urinary 8-isoprostanes was 180 pg/ml (IQR 157–192) while the one adjusted for creatinine was 0.13 ng/mg creatinine (IOR 0.08–0.23). The median unadjusted 8-OHdG was 5.3ng/mL (IQR 2.7–13.8) while adjustment for creatinine yielded a median of 4 ng/mg creatinine (IQR 2–10).

**Table 1:** Characteristics of enrolled participants in relation to markers of oxidative stress.

Variable	n	8-isoprostanes, ng/mg creatinine Median (IQR)	*p*-value	8-hydroxydeoxyguanosine, ng/mg creatinine Median (IQR)	*p*-value
Sex			0.07		0.08
Males	116	0.12 (0.08–0.21)		3.5 (1.6–8.0)	
Females	128	0.15 (0.08–0.25)		4.3 (2.4–11)	
Age group (years)			**0.007**		**0.01**
Less than 30	10	0.14 (0.10–0.28)		1.7 (1.3–4.3)	
30–44	89	0.11 (0.07–0.22)		3.7 (1.5–6.1)	
45–59	86	0.14 (0.09–0.21)		3.5 (2.1–12)	
60 and above	59	0.18 (0.10–0.39)		7.0 (2.7–17)	
Residence			0.78		0.71
Urban	199	0.12 (0.08–0.24)		3.7 (1.9–11)	
Rural	45	0.13 (0.08–0.20)		4.4 (2.4–8.0)	
Good accommodation*			0.65		0.40
Yes	199	0.12 (0.08–0.23)		4.0 (2.0–12)	
No	45	0.15 (0.09–0.23)		2.0 (4.4–6.8)	
Basic household goods**			0.78		0.80
Yes	217	0.14 (0.09–0.20)		3.9 (2.0–10)	
No	27	0.12 (0.08–0.24)		4.7 (2.1–10.7)	
Body mass index, kg/m^2^			0.28		0.05
Less than 18	14	0.12 (0.06–0.21)		8.5 (1.3–61)	
18–20	25	0.16 (0.10–0.24)		11 (2.7–21)	
21–24	55	0.11 (0.07–0.20)		3.1 (2.0–6.1)	
25–30	82	0.14 (0.09–0.25)		4.4 (2.3–11)	
Above 30	42	0.12 (0.07–0.22)		3.4 (1.3–6.4)	
HIV status			0.05		0.09
Positive	44	0.10 (0.06–0.17)		6.0 (2.0–20)	
Negative	176	0.14 (0.09–0.23)		3.5 (1.9–8)	
Smoking			0.52		0.90
Yes	12	0.11 (0.08–0.21)		3.3 (2-0-33)	
No	205	0.10 (0.08–0.21)		3.9 (1.9–9.6)	
Alcohol			**0.03**		0.32
Yes	58	0.10 (0.06–0.20)		4.5 (2.2–12)	
No	172	0.14 (0.09–0.25)		3.7 (1.8–9.3)	

*Residing in a brick house with a kitchen and piped water was considered as good accommodation.

**Being in possession of either a television, fridge, computer, car, cable connection or a microwave oven.

Using levels adjusted for urinary creatinine, 8-isoprostane and 8-OHdG no associations were found with body mass index, rural residence, smoking or HIV status. However, alcohol intake was associated with 8-isoprostane levels (Table 1).

### Association between markers of oxidative stress with dietary and environmental factors

**Fig 1:**
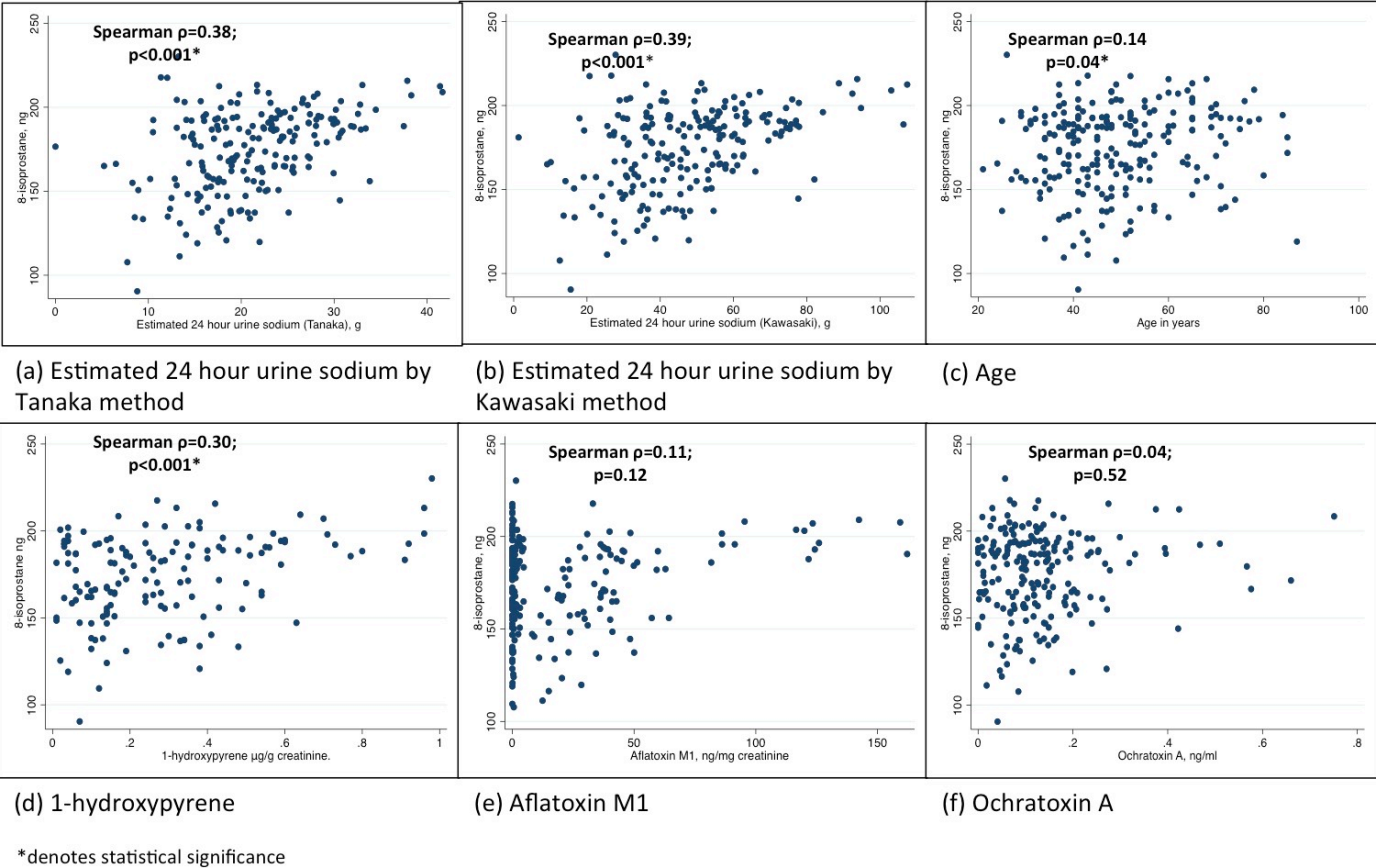
Correlations between urinary 8-isoprostanes levels and; (a) Estimated 24 hour urine sodium by Tanaka method, (b) Estimated 24 hour urine sodium by Kawasaki method (c) Age, (d) 1-hydroxypyrene, (e) Aflatoxin M1, (f) Ochratoxin A. *denotes statistical significance.

Associations between urinary levels of 8-isoprostane and age, BMI, estimated 24-hour urine sodium, 1-hydroxypyrene, aflatoxin M1 and ochratoxin A were evaluated by computing correlations. Significant correlations were observed with 1-hydroxypyrene (r = 0.30; *p*<0.001), and estimated 24-hour urine sodium by Tanaka (r = 0.38; *p*<0.001) and Kawasaki (r = 0.39; *p*<0.001) methods Fig 1. The median 8-isoprostane levels in individuals aged 60 years and above was 188pg/mL (IQR 166–198) while it was 175pg/mL (IQR 156–191) in those below 60 years.

Logistic regression analysis of dichotomised levels of 8-isoprostane and 8-OHdG showed that 1-hydroxypyrene and estimated 24-hour sodium were independent predictors of oxidative stress measured by creatinine adjusted and unadjusted 8-isoprostanes levels (Table 2). However, only unadjusted 8-OHdG was associated with 1-hydroxypyrene (*p* = 0.03) and age (*p* = 0.007; Table 2).

**Table 2:** Logistic regression for adjusted (a) and unadjusted (b) urinary 8-isoprostane dichotomized at the median.

**(a) 8-isoprostane adjusted for urine creatinine**	**Odds ratio**	**95% Confidence interval**	***p*-value**
1-hydroxypyrene, ng/mg creatinine	13.7	1.6–115	0.02
Estimated 24 hour urine sodium (Tanaka), g	1.1	1.05–1.2	0.003
**(b) Unadjusted 8-isoprostane**			
1-hydroxypyrene, ng/mg creatinine	162	8.5–301	0.001
Estimated 24 hour urine sodium (Tanaka), g	1.3	1.2–1.5	<0.001
**(c) Unadjusted 8-hydroxydeoxyguanosine**			
1-hydroxypyrene, ng/mg creatinine	12.5	1.8–85.7	0.01
Age in years	1.04	1.01–1.07	0.007

*Factors included in the regression model were 1-hydroxypyrene, body mass index, age, estimated 24-hour sodium excretion (by Tanaka methods), aflatoxin M1 and ochratoxin A

**Logistic regression model showing the output of significant results only.

### Analysis of 24-hour urine sodium and self reported salt ingestion

**Fig 2:**
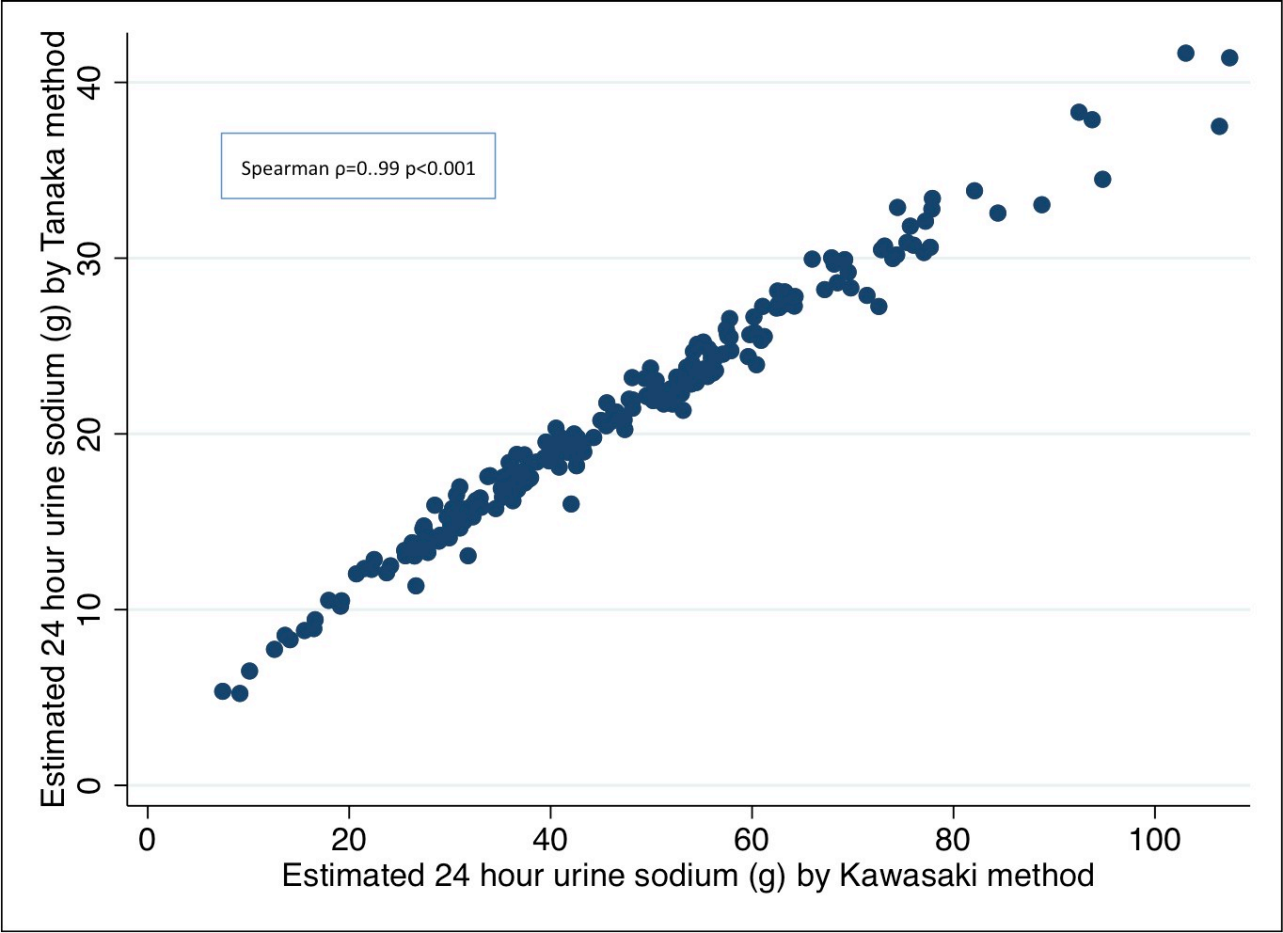
Correlation between the Tanaka and Kawasaki methods for estimation of 24-hour urine sodium from a single spot urine sample.

The Spearman correlation co-efficient between the Tanaka and Kawasaki methods for estimating 24-hour sodium was 0.99; *p*<0.001 (Fig 2).

The median 24-hour sodium excretion was 21 g (IQR 16–25 g), and all (100%) of the participants appeared to consume more sodium than the WHO recommended daily intake of 5 g. There was no association between 24-hour sodium excretion and sex (*p* = 0.38), age group (*p* = 0.25), education level (*p* = 0.78), residence (*p* = 0.31) or economic status (*p* = 0.48). However, there was an association between 24-hour urine sodium excretion and BMI. Participants with higher BMI had higher urinary sodium (r = 0.16; *p* = 0.02). Analysis using the Kawasaki method yielded similar results.

Using an interviewer-administered questionnaire, data on self reported salt intake and preference were collected. Of the 240 participants who gave complete information, 90 (37%) admitted to adding extra salt to already prepared food. And of these, 37/90 (41%) added it all the time, 32/90 (36%) very often and 21/90 (23%) not often. There was no association between these responses and the estimated salt excretion (*p* = 0.38). On salt preference, only 24 (10%) of the participants admitted to liking very salty food. Forty (16%) participants reported a dislike of salty food, while 41 (17%) and 134 (56%) preferred slightly salty and salty food respectively. Similarly, there was no association between reported salt preference and the estimated salt excretion, *p* = 0.54.

## Discussion

Long-term oxidative stress negatively impacts human health by increasing the risk of several non-communicable diseases (NCDs). We evaluated the association between oxidative stress and known risk factors for NCDs in Zambia. We found an association between urinary 8-isoprostane with estimated 24-hour urine sodium and exposure to polycyclic aromatic hydrocarbons.

Oxidative stress results from overproduction of reactive oxygen species (ROS), consequently causing damage to lipids, proteins and DNA [28]. It is thought that oxidative damage to DNA disrupts cellular function through somatic mutation, hence accelerating the ageing process [29]. Experiments in animal models have greatly contributed to our understanding of human physiological processes [30]. Clinical studies evaluating a direct link between oxidative stress and risk factors for NCDs are limited, and therefore many concepts have been derived from animal models. The influence of salt on oxidative stress for example, has been extensively studied in mice enabling us to understand that excess salt can enhance the production of ROS while impairing anti-oxidant mechanisms [10, 11]. There is evidence that high salt concentration causes DNA double stranded breaks, a process thought to contribute to genomic evolution [31]. Our study did not show a link between salt excretion and urinary evidence of DNA damage but there was a clear association with lipid oxidative stress. There is an urgent need to carry out more *in vivo* human experiments to demonstrate a direct link between salt consumption and oxidative stress.

All our study participants consumed much higher levels of salt than the WHO recommended maximum. This is consistent with the 2017 Zambia report on NCD risk factors, a community based country wide survey, which reported that the mean salt consumption was more than twice the WHO, recommended maximum [32]. That was despite the widely held impression by their respondents that they consumed appropriate amounts of salt. With the growing burden of NCDs, salt reduction has been identified as one of the most cost-effective measures for improvement of population health outcomes [13]. It is therefore important to disseminate the findings of studies such as this one as a contribution toward community wide sensitisation on the dangers of excess salt intake. However, study participants who admitted to adding extra salt or preferring very salty food did not necessarily have the highest sodium excretion. This finding highlights the need for sharper salt assessment approaches, the best being objective measurements. We estimated the salt intake from a single spot sample, and therefore individuals who took less salt the day before enrolment had less urinary sodium regardless of their past intake or preference. Measuring salt content of food taken over a period of time could have given a better picture of participant’s salt ingestion.

Over 70% of Africans rely on biomass fuels and are therefore constantly exposed to the smoke [33]. One of the major constituents of biomass smoke are polycyclic aromatic hydrocarbons (PAH). For PAH to exert metabolic effects they have to be bioactivated primarily by cytochrome P450 enzymes [34]. Smith et al., reported that PAH can lead to oxidative stress and alter the cellular redox status [35]. We measured 1-hydroxypyrene (1-OHP), a major PAH metabolite, which has also been used by other investigators to study the effects of exposure to biomass smoke [36]. In this study, we found an association between 1-OHP and oxidative stress. Some investigators reported similar associations in different disease states and occupations [37, 38], while others reported a link between oxidative damage to DNA and ambient air particulate matter [39].

In Zambia, studies evaluating aflatoxins have consistently reported high levels of food contamination [40, 41]. Generally, mycotoxins induce toxicity by direct cytotoxicity and/or generation of ROS with the resultant oxidative stress [42]. There is sufficient evidence linking aflatoxins to liver cancer and they are therefore identified as class 1 carcinogens [43]. Similarly, the effect of aflatoxins on health is evident in children with malnutrition particularly in sub-Saharan Africa [44, 45]. Evidence linking ochratoxins to carcinogenesis in humans is less well characterized [46]. Ochratoxins are however, known to cause oxidative stress by reducing antioxidant cellular defenses. They are nephrotoxic and have been associated with renal tumours [47]. Our results have not shown any link between oxidative stress and the two mycotoxins evaluated.

The lack of blood pressure measurements in this study could have confounded the results. With such high salt intakes, we would expect that many of these patients would have also been hypertensive. There is evidence that high salt intake leads to an impaired response of blood vessels to endothelium-dependent relaxations induced by a variety of vasodilator agents [48]. Some experimental models have also shown that high salt reduces nitric oxide levels by decreasing synthase activity, contributing to salt-sensitive hypertension [49]. Salt-sensitivity is associated with increased intracellular angiotensin II signal transduction [50].

Interpretation of our study results was limited by the fact that some of the assays we used measure short-term exposure. Estimated 24-hour urine sodium for example does not provide information about the overall salt intake of an individual. It varies depending on fluid consumption, time of the day, duration and volume of collection, participant’s posture, the time and amount of salt consumed in the last meal, as well as neural hormonal systems associated with cardiovascular outcomes [11]. Therefore, one measurement of a spot urine sample might not be enough to accurately measure of long-term exposure [51–53]. On the other hand, measurement of spot urine does serve as a good and reliable estimate in epidemiological studies such as this one [15–18]. In addition, our results have shown very high levels of estimated 24 hour urinary sodium. There will be need to further validate these findings in the same population using actual measurements of excreted sodium over 24 hours. Our reporting of markers oxidative stress is however reliable as we used assays that estimate long-term levels. Accurate and precise measurement of ROS is difficult due to their ephemeral nature and rapid reactivity [54]. Some investigators have attempted to determine normal reference ranges for biomarkers of oxidative stress [27, 28]. However, because there is no consensus on which levels of either 8-isoprostane or 8-OHdG should be considered normal or “stress free” [55], we elected to use raw values in our analyses as opposed to employing presumed cut-offs.

## Conclusion

This study has highlighted the importance of risk factors for NCDs in the occurrence of oxidative stress. It has demonstrated an association between oxidative stress and age, estimated 24-hour urine sodium and 1-hydroxypyrene in Zambian adults.
